# A Phase 3 Study of Ensitrelvir in Japanese Participants Aged 6-11 Years with SARS-CoV-2 Infection

**DOI:** 10.1093/jpids/piag071

**Published:** 2026-08-19

**Authors:** Takashi Nakano, Hiroyuki Moriuchi, Isao Miyairi, Takumi Imamura, Maria Matsumoto, Yuko Tsuge, Takeki Uehara

**Affiliations:** Department of Pediatrics, Kawasaki Medical School, Okayama, Japan; National Research Center for the Control and Prevention of Infectious Diseases, Nagasaki University, Nagasaki, Japan; Department of Pediatrics, Hamamatsu University School of Medicine, Hamamatsu, Shizuoka, Japan; Drug Development and Regulatory Science Division, Shionogi & Co., Ltd., Osaka, Japan; Drug Development and Regulatory Science Division, Shionogi & Co., Ltd., Osaka, Japan; Drug Development and Regulatory Science Division, Shionogi & Co., Ltd., Osaka, Japan; Drug Development and Regulatory Science Division, Shionogi & Co., Ltd., Osaka, Japan

**Keywords:** ensitrelvir, pediatric COVID-19, SARS-CoV-2 3CL protease inhibitor, pharmacokinetics, Japan

## Abstract

**Background:**

Children aged <12 years with coronavirus disease 2019 (COVID-19) have limited treatment options; currently approved oral antivirals are restricted by age or bodyweight. We evaluated the safety and plasma concentration-time profiles and exploratory efficacy of ensitrelvir, a once-daily oral 3C-like protease inhibitor, in children with mild-to-moderate COVID-19 in Japan.

**Methods:**

This phase 3, double-blind, placebo-controlled study randomized (2:1) participants aged 6 to <12 years presenting ≤72 h after COVID-19 onset to receive ensitrelvir (bodyweight-based dosing) or placebo once daily for 5 days. Primary endpoints were safety and pharmacokinetics. Secondary endpoints included change in viral ribonucleic acid (RNA) and time to resolution (TTR) of key COVID-19 symptoms (stuffy or runny nose and cough) plus fever.

**Results:**

Of 117 randomized participants (median age 10 years), 78 received ensitrelvir and 39 received placebo. Ensitrelvir was well tolerated with no new safety concerns. Plasma concentration-time profiles were similar across bodyweight categories (20 to <30, 30 to <40, and ≥40 kg). Ensitrelvir reduced viral RNA vs placebo on Day 4, by approximately 1.0 log_10_ copies/mL (95% CI, −1.45 to −0.47). Median TTR of key COVID-19 symptoms was 140.5 h with ensitrelvir and 146.2 h with placebo (median difference −5.7 h [95% CI, −73.6 to 43.4 h]).

**Conclusions:**

In children aged 6 to <12 years with mild-to-moderate COVID-19 treated with ensitrelvir, we observed a favorable safety profile, adult-matched plasma concentration-time profile, and a greater reduction in viral RNA vs placebo. Ensitrelvir represents a potential treatment option for underserved pediatric patients with mild-to-moderate COVID-19.

**Clinical Trial Registration:**

Japan Registry of Clinical Trials, jRCT2031230140 (https://jrct.mhlw.go.jp/en-latest-detail/jRCT2031230140).

## INTRODUCTION

The omicron variant of severe acute respiratory syndrome coronavirus 2 (SARS-CoV-2) emerged in late 2021, becoming epidemic in Japan by January 2022.^1^^,^^2^ The rates of pediatric coronavirus disease 2019 (COVID-19) spread in Japan were relatively stable pre-omicron, but markedly increased between 2021 and 2022, especially in children aged 5-14 years.^3–6^ Despite the availability of COVID-19 vaccines, uptake in this age group remains suboptimal,^7^ leaving many children vulnerable to SARS-CoV-2 infection.

Children and adults have a similar likelihood of contracting SARS-CoV-2,^8^ but children typically experience milder disease and are less likely to develop severe illness, require hospitalization, or develop post-COVID-19 condition (long COVID).^8–13^ Common symptoms in children include fever, cough, sore throat, headache, and rhinorrhea.^14^^,^^15^ Neurologic involvement occurred in ⁓12% of children with multisystem inflammatory syndrome.^16^^,^^17^ During Omicron variant waves in Japan, 32 and 68 cases of SARS-CoV-2-associated encephalopathy were identified in children during the BA.1/BA.2 and the BA.5 predominant periods, respectively.^18^ In Asia, mortality rates reached up to 66.7% in these patients.^18–21^

In Japan, remdesivir (intravenous injection) is approved for children aged <12 years with COVID-19 and weighing from 3.5 to 40 kg.^22^ Furthermore, ritonavir-boosted nirmatrelvir recently received approval in Japan for children aged ≥6 years with COVID-19 and weighing ≥20 kg.^23^ As of April 2026, molnupiravir has not been approved in Japan for patients aged <12 years with COVID-19.^24^

Ensitrelvir fumaric acid (hereafter “ensitrelvir”) is a novel, once-daily, oral 3C-like protease (3CL^pro^) inhibitor that prevents viral replication.^25^ Based on the positive findings of the registrational phase 2/3 SCORPIO-SR study conducted in Japan, South Korea, and Vietnam,^26–28^ ensitrelvir received full approval in Japan to treat patients aged ≥12 years with mild-to-moderate COVID-19, emergency approval in Singapore, and fast-track designation from the United States Food and Drug Administration.^29–31^

A phase 3 trial was conducted in Japan to investigate the safety and pharmacokinetic (PK; plasma concentration-time) profiles of ensitrelvir in children aged 6 to <12 years with mild-to-moderate COVID-19.

## METHODS

### Study Design and Participants

Details of the study design, enrollment criteria, and bodyweight-based dosing are provided in Supplementary Methods and Table S1. In brief, this phase 3, multicenter, randomized, double-blind, placebo-controlled study (jRCT2031230140)^32^ enrolled participants aged 6 to <12 years with SARS-CoV-2 infection and mild-to-moderate COVID-19 from 43 sites across Japan. Participants were grouped by Day 1 bodyweight (20 to <30 kg, 30 to <40 kg, and ≥40 kg); each group was randomized 2:1 (using the minimization method) to receive either ensitrelvir or placebo once daily for 5 days (Days 1-5; Table S1). Placebo and ensitrelvir tablets were indistinguishable in appearance. Randomization was stratified using the minimization method with SARS-CoV-2 primary vaccination status (completion vs non-completion) and bodyweight. Participants were followed for 28 days after the first intervention dose (Figure S1).

Eligible patients were aged 6 to <12 years and weighed ≥20 kg, with confirmed SARS-CoV-2 infection and COVID-19 onset (Table S2) within 72 h before randomization and had ≥1 moderate or severe COVID-19 symptom among 12, as listed in Table S2 at screening. Requirement for supplemental oxygen or mechanical ventilation; COVID-19-associated MIS-C/PIMS; or prior receipt of ensitrelvir were exclusionary (Table S3).

This study followed the ethical principles of international guidelines, including the Declaration of Helsinki,^33^ Council for International Organizations of Medical Sciences International Ethical Guidelines,^34^ Good Clinical Practice guidelines,^35^ and applicable laws and regulations. The protocol and informed consent forms were approved by an institutional review board before trial initiation. All parties were blinded to the study intervention, including analysis of cohort 1 (Figure S1). All participants (or legal representatives) provided written informed consent.

### Objectives and Endpoints

Primary objectives were to confirm the safety, tolerability, and plasma concentration-time profiles of ensitrelvir once daily (QD) × 5 in pediatric patients infected with SARS-CoV-2. Primary endpoints included adverse events (AEs), laboratory tests, vital signs, and plasma ensitrelvir concentration.

Secondary objectives compared the efficacy and antiviral effect of ensitrelvir vs placebo. Secondary endpoints included change from baseline to Day 4 in SARS-CoV-2 viral ribonucleic acid (RNA) and titer; change from baseline in viral titer at each timepoint (Days 2, 4, 6, 14, and 21); time to resolution (TTR) of 2 COVID-19 objective symptoms (stuffy/runny nose and cough) plus fever; and TTR of 5, 12, and 14 common COVID-19 symptoms (Table S2; definitions of symptom resolution are provided in Supplementary Methods).

Exploratory objectives included changes from baseline in non-SARS-CoV-2 respiratory pathogens at each timepoint (Days 2, 4, 6, 14, and 21) and evaluation of treatment-emergent amino acid substitutions (TEAASs) in the gene encoding 3CL^pro^ (*nsp5*) on Day 1 and post-ensitrelvir administration.

### Study Procedures

Study procedure scheduling is provided in Table S4. AEs were assessed continuously; laboratory tests and blood and nasal-swab sampling were conducted at prespecified timepoints throughout the treatment and follow-up periods. The method for evaluating taste and smell disorders is described in Supplementary Methods and Table S2. Viral RNA were measured in nasal-swab samples using reverse transcription-polymerase chain reaction. TEAASs were detected by next-generation sequencing of *nsp5*. COVID-19 symptoms were diarized by participants or their legal representatives.

### Statistical Analysis

The details of sample size determination, analysis populations, and statistical analysis methods are provided in Supplementary Methods and Tables 5 and 6, respectively. In brief, participant-level AEs (safety analysis population) were analyzed descriptively. Treatment-emergent AEs (TEAEs) were summarized by severity, study drug relatedness (treatment-related AEs [TRAEs]), and age group (6-8 years and 9-11 years). PK data (PK analysis population) were estimated separately for 3 bodyweight categories using population PK parameters derived from data for an older population (age 12 to <70 years), in participants with SARS-CoV-2 infection and in healthy adults.^36^ Plasma concentrations of ensitrelvir 24 h after administration (C_24_) on Days 1 and 5 and 4 h after administration (C_4_) on Days 4 and 5 are summarized as numbers, mean, and standard deviation (SD). PK analysis population was defined as all participants who received at least 1 dose of ensitrelvir and had at least 1 evaluable plasma concentration. Viral response and TTR were analyzed in the intention-to-treat (ITT) populations. Efficacy parameters were analyzed by assigned treatment arm. TTR was analyzed descriptively; comparisons of change from baseline in viral load between the ensitrelvir and placebo arms were performed using analysis of covariance. No adjustment was made for multiplicity. Sites of detected polymorphic substitutions in *nsp5* TEAASs (ITT population) were recorded.

Statistical analyses were conducted using SAS software version 9.4 or higher (SAS Institute, Cary, NC, USA) and Phoenix WinNonlin software version 8.3 (Certara, Radnor, PA, USA).

## RESULTS

### Participants Disposition and Baseline Characteristics

Of 117 randomized and treated participants (ensitrelvir, *n* = 78; placebo, *n* = 39), 115 (98%) completed the study (Figure S2). The reasons for study discontinuation were consent withdrawal (*n* = 1, ensitrelvir arm) and the investigator’s decision due to participant burden (*n* = 1, placebo arm). Baseline participant characteristics were generally well balanced between the 2 arms (Table 1). Overall, median age was 10 years (range, 6-11 years); most participants (91%) initiated study treatment ≤48 h post-onset of COVID-19 symptoms, and 41% had received ≥1 SARS-CoV-2 vaccination.

**Table 1:** Participant Demographic and Clinical Characteristics by Bodyweight Category (ITT Population)

Data are *n* (%) unless stated otherwise	Ensitrelvir arm				Placebo arm			
	20 to <30 kg *n* = 31	30 to <40 kg *n* = 28	≥40 kg *n* = 18	All participants *N* = 77	20 to <30 kg *n* = 15	30 to <40 kg *n* = 13	≥40 kg *n* = 9	All participants *N* = 37
**Sex**								
Male	15 (48)	14 (50)	12 (67)	41 (53)	4 (27)	10 (77)	3 (33)	17 (46)
Female	16 (52)	14 (50)	6 (33)	36 (47)	11 (73)	3 (23)	6 (67)	20 (54)
**Age (years) at signed ICF**								
*N*	31	28	18	77	15	13	9	37
Mean (SD)	7.9 (1.6)	10.1 (1.0)	10.5 (0.6)	9.3 (1.7)	8.1 (1.5)	10.0 (1.2)	10.8 (0.7)	9.4 (1.6)
Median	8.0	10.0	11.0	10.0	8.0	10.0	11.0	10.0
Range	6-11	8-11	9-11	6-11	6-11	7-11	9-11	6-11
**Time from onset to first study intervention, h**								
≤24	15 (48)	12 (43)	9 (50)	36 (47)	7 (47)	7 (54)	3 (33)	17 (46)
>24 to ≤48	14 (45)	14 (50)	7 (39)	35 (45)	5 (33)	5 (38)	6 (67)	16 (43)
>48 to ≤72	2 (6)	2 (7)	2 (11)	6 (8)	3 (20)	1 (8)	0	4 (11)
>72	0	0	0	0	0	0	0	0
**SARS-CoV-2 vaccination**								
Yes	10 (32)	10 (36)	12 (67)	32 (42)	5 (33)	5 (38)	5 (56)	15 (41)
No	21 (68)	18 (64)	6 (33)	45 (58)	10 (67)	8 (62)	4 (44)	22 (59)
**Number of SARS-CoV-2 vaccinations received**								
0	21 (68)	18 (64)	6 (33)	45 (58)	10 (67)	8 (62)	4 (44)	22 (59)
1	1 (3)	1 (4)	0	2 (3)	2 (13)	0	0	2 (5)
2	5 (16)	7 (25)	2 (11)	14 (18)	0	3 (23)	2 (22)	5 (14)
3	3 (10)	1 (4)	8 (44)	12 (16)	3 (20)	1 (8)	1 (11)	5 (14)
≥4	1 (3)	1 (4)	2 (11)	4 (5)	0	1 (8)	2 (22)	3 (8)

Abbreviations: ICF = informed consent form; ITT = intention-to-treat; SARS-CoV-2 = severe acute respiratory syndrome coronavirus 2; SD = standard deviation.

### Safety

Post-study drug administration, 41% of participants in the ensitrelvir arm (*n* = 32/78) and 31% in the placebo arm (*n* = 12/39) experienced ≥1 TEAE (Table 2).

**Table 2:** Summary of TEAEs and list of TEAEs (by preferred term) occurring in ≥2 participants in either treatment arm, by severity (safety analysis population)

Data are *n* (%) unless stated otherwise	Ensitrelvir arm				Placebo arm			
	20 to <30 kg *n* = 31	30 to <40 kg *n* = 29	≥40 kg *n* = 18	All participants *N* = 78	20 to <30 kg *n* = 16	30 to <40 kg *n* = 14	≥40 kg *n* = 9	All participants *N* = 39
**TEAEs** ^a^								
Events, *n*	16	14	9	39	4	6	3	13
Participants	14 (45)	11 (38)	7 (39)	32 (41)	4 (25)	5 (36)	3 (33)	12 (31)
95% CI (%)^b^	27-64	21-58	17-64	30-53	7-52	13-65	8-70	17-48
**Any TEAEs**								
Moderate	2 (6)	2 (7)	2 (11)	6 (8)	0	0	0	0
Mild	12 (39)	9 (31)	5 (28)	26 (33)	4 (25)	5 (36)	3 (33)	12 (31)
**TEAEs (by preferred term)**								
Influenza (any)	1 (3)	3 (10)	2 (11)	6 (8)	0	0	0	0
Moderate	1 (3)	2 (7)	1 (6)	4 (5)	0	0	0	0
Mild	0	1 (3)	1 (6)	2 (3)	0	0	0	0
Nasopharyngitis (any)	2 (6)	0	0	2 (3)	0	0	0	0
Moderate	1 (3)	0	0	1 (1)	0	0	0	0
Mild	1 (3)	0	0	1 (1)	0	0	0	0
Headache (any)	1 (3)	0	0	1 (1)	0	1 (7)	1 (11)	2 (5)
Moderate	0	0	0	0	0	0	0	0
Mild	1 (3)	0	0	1 (1)	0	1 (7)	1 (11)	2 (5)
Vomiting (any)	3 (10)	0	0	3 (4)	0	0	0	0
Moderate	0	0	0	0	0	0	0	0
Mild	3 (10)	0	0	3 (4)	0	0	0	0
Diarrhea (any)	0	1 (3)	0	1 (1)	1 (6)	0	1 (11)	2 (5)
Moderate	0	0	0	0	0	0	0	0
Mild	0	1 (3)	0	1 (1)	1 (6)	0	1 (11)	2 (5)
HDL decreased (any)	3 (10)	5 (17)	3 (17)	11 (14)	0	0	0	0
Moderate	0	0	0	0	0	0	0	0
Mild	3 (10)	5 (17)	3 (17)	11 (14)	0	0	0	0
Blood TG increased (any)	0	0	0	0	0	1 (7)	1 (11)	2 (5)
Moderate	0	0	0	0	0	0	0	0
Mild	0	0	0	0	0	1 (7)	1 (11)	2 (5)

^a^Classified according to the Medical Dictionary for Regulatory Activities version 25.1.

^b^Calculated using the Clopper-Pearson method. Abbreviations: CI = confidence interval; HDL = high-density lipoprotein; TEAE = treatment-emergent adverse event; TG = triglyceride.

No serious TEAEs, deaths, or TEAE-related treatment discontinuations occurred. TEAEs were predominantly mild, and most had resolved by the end of follow-up; moderate TEAEs occurred exclusively in the ensitrelvir arm (Table 2).

TRAEs occurred in 19% (ensitrelvir) and 3% (placebo) of participants; all were mild and had resolved by Day 28. The most frequent ensitrelvir TREAs were decreased high-density lipoprotein (HDL; 14%) and vomiting (4%). Diarrhea was the only TRAE reported with placebo (*n* = 1).

### Plasma Concentration-Time Profile

**Figure 1:**
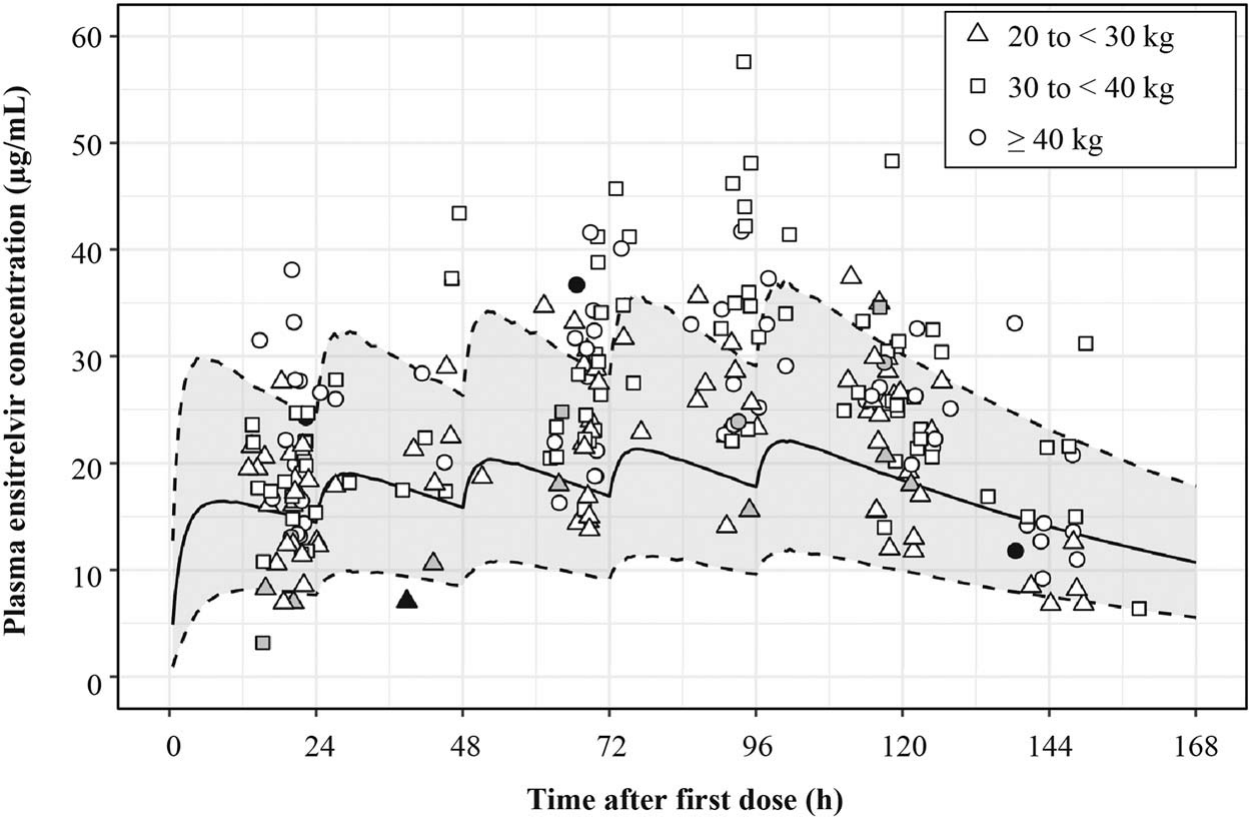
Observed Plasma Concentrations of Ensitrelvir Over Time during Treatment in Pediatric Participants (PK Analysis Population, *n* = 78). Solid line: mean of adults based on PPK analysis. Break line: Upper and lower estimation of 5% of adults based on PPK analysis. Black filled symbols = participants with no study drug administration at the previous timepoint. Gray-filled symbols = participants with partial drug administration at the previous timepoint. Abbreviations: PK = pharmacokinetic; PPK = process performance index.

Ensitrelvir exposure over the 5-day treatment period and 2 days of follow-up in the PK-evaluable population (*n* = 78) is shown in Figure 1. There were no notable differences in plasma concentration-time profile for ensitrelvir between bodyweight categories (Table S7), and no notable differences in PK were observed between age groups or by sex (data not shown).

### SARS-CoV-2 Viral RNA and Viral Titer

The reduction in SARS-CoV-2 viral RNA between baseline and Day 4 was greater with ensitrelvir vs placebo (difference [95% confidence interval (CI)] in least-squares mean [LSM]: −0.96 log_10_ copies/mL [−1.45 to −0.47 log_10_ copies/mL]; Table 3). The reduction from baseline in SARS-CoV-2 viral titer on Day 2 was also greater in the ensitrelvir arm (difference [95% CI] in LSM vs placebo: −0.39 log_10_ 50% tissue culture infectious dose [TCID_50_]/mL [−0.69 to −0.08 log_10_ TCID_50_/mL]; Table S8).

**Table 3:** Change in SARS-CoV-2 Viral RNA (by RT-PCR)^a^ on Day 4 in Participants Treated with Ensitrelvir or Placebo (ITT Population)

Viral RNA (log _10_ copies/mL)	Ensitrelvir arm *N* = 77	Placebo arm *N* = 37
**Baseline**		
*n*	76	37
Mean (SD)	6.47 (1.22)	6.54 (1.17)
**Day 4**		
*n*	74	36
Mean (SD)	−3.34 (1.23)	−2.39 (1.26)
Median	−3.20	−2.32
Min, max	−7.50, 1.08	−5.35, 0.07
ANCOVA vs placebo^b^		
LSM (SE)	−3.34 (0.14)	−2.38 (0.20)
Difference in LSM (SE)	−0.96 (0.25)	−
95% CI for difference	−1.45 to −0.47	−
Two-sided *P*-value^c^	.0002	−

^a^LLOQ of viral RNA was 2.08 log_10_ copies/mL. If viral RNA was negative and less than the LLOQ, the viral RNA was imputed as 0 and 1.97 log_10_ copies/mL, respectively.

^b^Covariate: SARS-CoV-2 viral RNA at baseline.

^c^ANCOVA.

Abbreviations: ANCOVA = analysis of covariance; CI = confidence interval; ITT = intention-to-treat; LLOQ = lower limit of quantification; LSM = least-squares mean; max = maximum; min, minimum; RNA = ribonucleic acid; RT-PCR = reverse transcription-polymerase chain reaction; SARS-CoV-2 = severe acute respiratory syndrome coronavirus 2; SD = standard deviation; SE = standard error.

### T‌TR of COVID-19 Symptoms

Median TTR (mTTR) of 2 COVID-19 objective symptoms (stuffy/runny nose and cough) plus fever was 140.5 h with ensitrelvir and 146.2 h with placebo (median difference [95% CI], −5.7 h [−73.6 to 43.4 h]; Figure S3A, Table S9). mTTR for these symptoms was 153.5 h with ensitrelvir and 176.9 h with placebo in participants with ≥3 moderate (or worse) COVID-19 symptoms at baseline (median difference [95% CI]: −23.4 h [−172.2 to 161.1 h]); it was 140.5 h with ensitrelvir and 134.0 h with placebo in participants with ≤2 symptoms (median difference [95% CI]: 6.5 h [−73.4 to 68.9 h]; Figure S3B and C; Table S10).

In participants with 5 COVID-19 symptoms, mTTR was similar between the 2 treatment arms (median [95% CI] difference: −0.8 [−62.4 to 41.7 h]; Table S9). mTTR was 144.7 h with ensitrelvir and 180.7 h with placebo in those with ≥3 moderate (or worse) COVID-19 symptoms at baseline (median difference [95% CI]: −36.1 h [−150.3 to 101.4 h]). It was 136.9 h with ensitrelvir and 130.2 h with placebo in those with ≤2 symptoms (median difference [95% CI]: 6.8 h [−35.6 to 78.4 h]; Table S10).

Kaplan–Meier curves of TTR for participants with 2 COVID-19 objective symptoms plus fever (Figure S3A-C) and those with 5 COVID-19 symptoms (data not shown) overall or with ≥3 moderate (or worse) symptoms at baseline suggest that from around 216-240 h post-treatment initiation, proportionately more placebo- vs ensitrelvir-treated participants experienced symptom resolution.

The pattern was similar for participants with 12 or 14 COVID-19 symptoms compared with 5 symptoms, whereby mTTR was similar for ensitrelvir- and placebo-treated participants (median [95% CI] difference: 2.7 h [−73.3 to 52.1 h] for both; Table S9). Among participants with 12 COVID-19 symptoms, it was 157.5 h with ensitrelvir and 186.4 h with placebo in the subgroup with ≥3 moderate (or worse) COVID-19 symptoms at baseline (median difference [95% CI]: −28.8 h [−174.9 to 96.2 h]); among those with ≤2 symptoms it was 143.1 h with ensitrelvir and 130.2 h with placebo (median difference [95% CI]: 12.9 h [−64.4 to 84.9 h]; Table S10).

### Respiratory Pathogens

At baseline, respiratory pathogens were detected in 6/77 (8%) evaluable participants in the ensitrelvir arm and 5/37 (14%) in the placebo arm (Table S11). By Day 21, these proportions had increased to 21% (*n* = 16/76) in the ensitrelvir arm and declined to 3% (*n* = 1/36) in the placebo arm (Table S12). The trend was consistent in the subgroup with ≥3 moderate (or worse) COVID-19 symptoms at baseline, with proportions on Day 21 of 19% and 6%, respectively. Among those with ≤2 moderate (or worse) COVID-19 symptoms, proportions fluctuated over time in both arms but were 21% vs 0%, respectively, at Day 21 (Table S12).

### 3CL^pro^ TEAASs

Nasal swabs from 27 of 77 ensitrelvir-treated participants met the criteria for next-generation sequencing analysis (>4.0 log_10_ copies/mL in viral RNA). TEAASs were detected in 3 (11%) participants: M49L, P96H, and C128R in one participant each.

## DISCUSSION

The present findings provide important evidence supporting the safety and plasma concentration-time profiles, antiviral efficacy, and clinical benefit of oral ensitrelvir for the treatment of mild-to-moderate COVID-19 in children aged 6 to <12 years. The safety profile of ensitrelvir in this population was manageable and consistent with the known safety profile for adolescents and adults,^26–28^ with no new safety concerns irrespective of baseline bodyweight.

All TEAEs were transient and mild or moderate, and none were serious or caused treatment discontinuation or death. TEAE incidence was higher with ensitrelvir than with placebo in participants weighing 20 to <30 kg, and comparable between the 2 arms for the other 2 bodyweight categories. TEAE incidence in the ensitrelvir arm was similar to that for participants aged 12 to <70 years in the ensitrelvir 375/125 mg arm of SCORPIO-SR (41% vs 44%), while TRAE frequency was lower (19% vs 25%).^28^ The rates of treatment-related decreased HDL, one of the main AEs associated with ensitrelvir,^30^ were similar between the present ensitrelvir arm and the SCORPIO-SR ensitrelvir 375/125 mg arm (14% vs 18%).^28^ Key TEAEs and/or TRAEs reported at higher rates in ensitrelvir-treated adolescents and adults vs their placebo-treated counterparts, such as triglyceride or bilirubin elevations and dyslipidemia (excluding increased HDL),^28^ were not observed in the present ensitrelvir-treated pediatric population.

The ensitrelvir plasma concentration-time profile showed no notable differences across bodyweight categories. Plasma ensitrelvir concentration in children weighing ≥20 kg was generally ≤90% of the predicted range (8.4-46.8 μg/mL, 125 mg dose, participants aged ≥12 years [SCORPIO-SR]).^36^ Even at concentrations ≥90% of the predicted range for the approved adult dose (125 mg),^30^ plasma ensitrelvir concentrations did not exceed those reported for adults/adolescents who received high doses (250 mg).^36^ Exposure of ensitrelvir was slightly higher in participants weighing 30 to <40 kg, where the dose per body weight was higher, but it did not exceed that of the high-dose group (250 mg/750 mg) in the adult SCOPIO-SR trial.^28^ Body weight can influence the PK of ensitrelvir.^36^ These findings suggest that the safety and effectiveness of ensitrelvir in adult patients can also be expected in their pediatric counterparts, and that the dosage regimen used in this study was appropriate for pediatric patients and achieved antiviral efficacy without exceeding safety thresholds.

Ensitrelvir administered once daily for 5 days alleviated COVID-19 symptoms in pediatric participants, particularly those with worse symptoms at baseline, showing reduced mTTR of symptoms compared with placebo. According to Kaplan–Meier estimates, the proportions of participants with resolution of 2 COVID-19 objective symptoms plus fever and of 5 symptoms (data not shown) were higher with ensitrelvir vs placebo for up to 240 h (10 days) post-treatment initiation and thereafter proportionately more placebo-treated participants experienced symptom resolution. Some possible explanations for the lack of symptom resolution by Day 21 include a smaller sample size compared with the symptom resolution demonstrated in a larger sample size in the SCORPIO-SR^28^ study, and a subsequent infection with another respiratory pathogen during the observation period (Table S12). mTTR of 5 predefined symptoms was shorter in this pediatric population (140.5 h [95% CI, 109.4-168.0]) than in participants aged 12 to <70 years with mild-to-moderate COVID-19 < 72 h from onset in SCORPIO-SR (167.9 h [95% CI, 145.0-197.6])^28^ and adults (aged ≥18 years) in SCORPIO-HR (7.0 days [168 h]; interquartile range, 4.0 to 14.0 days [96-336 h]).^37^ In the present placebo arm, mTTR was also shorter than in the SCORPIO-SR placebo population (146.2 h [95% CI, 92.0-188.9 h] vs 192.2 h [95% CI, 174.5-283.3]).^28^

Ensitrelvir antiviral activity in this study was comparable with that reported for ensitrelvir-treated participants aged 12 to <70 years, with reductions in both SARS-CoV-2 viral RNA levels and viral titer compared with placebo,^28^ and eliciting greater reductions in both variables vs placebo by Day 4 and Day 2, respectively. The ensitrelvir-induced mean (SD) change in viral RNA (log_10_ copies/mL) from baseline (Day 4: ensitrelvir, −3.34 [1.23] vs placebo, −2.39 [1.26]) was greater than that reported in SCORPIO-SR (LSM [standard error] change from baseline, Day 4: ensitrelvir, −2.48 [0.08] vs placebo, −1.01 [0.08] for the 125-mg dose).^28^ Although a direct comparison is difficult due to different viral variants, ensitrelvir administration resulted in a greater reduction in viral RNA levels than placebo in both adult and pediatric patients. The observed consistency between the ensitrelvir antiviral effects and the plasma concentration-time profile in the present study was greater than that reported for adults.^36^

Resistance to antivirals remains a concern. TEAASs were detected in just 3 of 27 children treated with ensitrelvir, only one of which, M49L, had known reduced susceptibility to ensitrelvir.^30^^,^^38^ Although the small sample size limits definitive conclusions, the low rate and limited impact of these mutations suggest that short-term treatment with ensitrelvir is unlikely to confer meaningful resistance.

Study strengths include its multicenter, randomized, double-blind, placebo-controlled design, which minimizes bias, helps to establish causality, and enhances generalizability to the broader population. The findings provide a comprehensive evaluation of the safety, PK, antiviral activity, and clinical outcomes of ensitrelvir, including by baseline symptom severity. Dosing tailored to 3 pediatric bodyweight categories was confirmed to account for growth-related changes in body composition and development. The study is limited by its small sample size and geographic restriction to Japan. History of SARS-CoV-2 infection data was not collected, as many SARS-CoV-2-infected children were likely to be asymptomatic or had only mild symptoms, and therefore did not seek medical consultation. Larger studies are essential to confirm the findings of this study. Furthermore, studies of ensitrelvir effectiveness according to vaccination history and against emerging SARS-CoV-2 variants may provide broader insights.

## CONCLUSIONS

Ensitrelvir was well tolerated in children with mild-to-moderate COVID-19 aged 6 to <12 years and achieved PK exposures that were at least comparable with those measured in adults, with a meaningful antiviral effect. Regarding symptom resolution, no difference between ensitrelvir and placebo was observed in the total population, whereas signs of symptom improvement were observed among participants with ≥3 moderate (or worse) COVID-19 symptoms at disease onset. Thus, ensitrelvir can be a promising therapeutic option for COVID-19 treatment in children.

## Supplementary Material

Supplement_SCORPIO_PED_Supplementary_Methods_Tables_S1-S12_Figures_S1-S3_piag071

## Data Availability

Shionogi & Co., Ltd. is committed to disclosing the synopses and results of its clinical trials and sharing clinical trial data with researchers upon reasonable request.
