# Supplementary material for: A Phase 3 Study of Ensitrelvir in Japanese Participants Aged 6-11 Years with SARS-CoV-2 Infection
Source: J Pediatric Infect Dis Soc. 2026 Aug 19;15(8):piag071. doi: 10.1093/jpids/piag071 (PMC13526401; doi:10.1093/jpids/piag071)
Supplement: Supplementary file 1 [file supplement_scorpio_ped_supplementary_methods_tables_s1-s12_figures_s1-s3_piag071.docx]

SUPPLEMENTARY INFORMATION

A phase 3 study of ensitrelvir in Japanese participants aged 6–11 years
with SARS-CoV-2 infection

Takashi Nakano, Hiroyuki Moriuchi, Isao Miyairi, Takumi Imamura, Maria Matsumoto, Yuko Tsuge, Takeki Uehara

**Table of Contents**

[SUPPLEMENTARY METHODS	4](#_Toc238127753)

[Enrollment schema	4](#_Toc238127754)

[Table S1 Weight-based dosing of ensitrelvir and placebo	5](#_Toc238127755)

[Statistical analysis	6](#_Toc238127756)

[Sample size determination	6](#_Toc238127757)

[PK analysis	6](#_Toc238127758)

[Efficacy analysis	6](#_Toc238127759)

[**Figure S1** (**A**) Schema of the study cohorts. (**B**) Study design.	8](#_Toc238127760)

[**Figure S2** Participant disposition.	9](#_Toc238127761)

[**Figure S3** K–M plots of TTR of two COVID-19 objective symptoms (stuffy/ runny nose and cough plus fever) (**A**) overall, and in participants with (**B**) ≥3 and (**C**) ≤2 moderate (or worse) COVID-19 symptoms at baseline (ITT population).	10](#_Toc238127762)

[Table S2 Typical COVID-19 symptoms grouped as evaluated in this study	11](#_Toc238127763)

[Table S3 Study eligibility criteria	13](#_Toc238127764)

[Definitions of symptom resolution	18](#_Toc238127765)

[Table S4 Schedule of laboratory tests and tissue sampling	20](#_Toc238127766)

[Table S5 Definition of study analysis populations	22](#_Toc238127767)

[Table S6 Statistical methods used to analyze selected secondary efficacy endpoints	23](#_Toc238127768)

[SUPPLEMENTARY RESULTS	25](#_Toc238127769)

[Table S7 Plasma concentrations of ensitrelvir (PK analysis population)	25](#_Toc238127770)

[Table S8 Change from baseline in SARS-CoV-2 viral titer^a^ on Day 2 and Day 4 (modified ITT population)	27](#_Toc238127771)

[Table S9 TTR of COVID-19 symptoms in participants who were SARS-CoV-2 positive at baseline by RT-PCR and who had onset of COVID-19 within 72 h before randomization (ITT population)	29](#_Toc238127772)

[Table S10 TTR of COVID-19 symptoms according to number of moderate (or worse) COVID-19 symptoms (≥3 or ≤2) at baseline (ITT population)	31](#_Toc238127773)

[Table S11 Respiratory pathogens detected in the study (ITT population)	33](#_Toc238127774)

[Table S12 Proportion of participants in whom non-SARS-CoV-2 respiratory pathogens were detected over time (irrespective of the presence of SARS-CoV-2 infection; ITT population)	35](#_Toc238127775)

[SUPPLEMENTARY REFERENCES	36](#_Toc238127776)

# SUPPLEMENTARY METHODS

## Enrollment schema

Ensitrelvir was dosed based on Day 1 bodyweight, as shown in Supplementary Table 1. The appropriateness of ensitrelvir dosing was first confirmed by the Safety Review Committee’s evaluation of the safety and pharmacokinetic (PK) data to Day 28 of the first 20 participants enrolled in the 20 to <30 kg and 30 to <40 kg bodyweight categories (cohort 1); enrollment to all three bodyweight categories was allowed thereafter (cohort 2; Supplementary Figure 1A). The safety of ensitrelvir in individuals aged ≥12 years and weighing ≥40 kg had already been confirmed in Study 2108T1221;^1^ therefore, participants weighing ≥40 kg were not enrolled in cohort 1 in this study. However, in case of any significant departure in cohort 1 from the predicted plasma concentration-time profile, the dose was changed depending on the PK data review.

**Virologic Assessments:**

Nasopharyngeal swabs collected from participants by the investigator on Days 1, 2, 4, and 6 (allowable width +1 day) and on Days 14, 21, and 28 (allowable width up to ±3 days) were used to measure SARS-CoV-2 viral RNA levels and viral titers. SARS-CoV-2 viral titers and viral RNA levels were centrally measured at Cerba Research (Rotterdam, Netherlands).

Briefly, viral RNA levels were measured using quantitative SARS-CoV-2 real-time PCR in duplex with the internal control consisting of Phocine Distemper Virus cell culture isolate. A four-point standard curve with a known number of SARS-CoV-2 RNA fragments (log_10_ copies/mL) was run together with every real-time PCR. Ct values are converted to log_10_ copies/mL based on the slope and intercept of the standard curve accompanying that run. Viral titers were determined based on a virus titration assay using VeroE6/TMPRSS2 cells. The results of the titration are transformed into log_10_ TCID_50_/mL.

### Table S1 Weight-based dosing of ensitrelvir and placebo

| **Bodyweight on Day 1** | **Dose (ensitrelvir or placebo)** | |
| --- | --- | --- |
|  | **Day 1** | **Days 2–5** |
| **≥40 kg** | 375 mg  (3×125-mg tablet) | 125 mg  (1×125-mg tablet) |
| **30 to <40 kg** | 250 mg  (2×125-mg tablet) | 125 mg  (1×125-mg tablet) |
| **20 to <30 kg** | 150 mg  (6×25-mg tablet) | 75 mg  (3×25-mg tablet) |

## Statistical analysis

### Sample size determination

A minimum sample size in the ensitrelvir arm that ensured a 90% probability of observing an adverse event with an expected incidence of 3% in at least one participant was calculated to be 76; the target sample size (assuming 5% attrition) was thus 80 for the ensitrelvir arm and 40 for the placebo arm (with a 2:1 assignment ratio).

### PK analysis

Pediatric PK data (PK analysis population) were estimated for each of the three weight categories using population PK parameters calculated using the results for an older population (age 12 to <70 years) from a phase 1 study and a phase 2/3 study in healthy adults,^2^ assuming that clearance was proportional to weight and volume of distribution to weight raised to the powers of 0.75 and 1, respectively. Based on these predictions, the dose for each weight category was proposed such that the associated exposure (maximum plasma concentration and area under the time–concentration curve) was similar to that after administration of 375/125 mg ensitrelvir to adults. For all bodyweight categories, the plasma concentration of ensitrelvir at 24 h after administration on Days 2 and 6 and at 4 h after administration on Days 4 and 5 are summarized as *n*, mean, standard deviation, coefficient of variation (CV), geometric mean and its CV, and median, minimum, and maximum values; when possible, PK data were also summarized by age group and sex.

### Efficacy analysis

Analysis of variance was used to compare viral load data between the treatment arms. For time to resolution (TTR) of coronavirus disease 2019 (COVID-19) symptoms, median (95% confidence interval) was calculated, and comparison of ensitrelvir vs placebo was conducted using the log-rank test and Peto–Prentice’s generalized Wilcoxon test, with hazard ratios estimated using Cox proportional hazards. Subgroup analysis for TTR of two COVID-19 symptoms plus fever was performed according to the number of moderate or worse COVID-19 symptoms at baseline (≤2 symptoms and ≥3 symptoms). Across efficacy data, the Kaplan–Meier method was used to analyze time-to-event data, and estimated restricted mean survival time with a follow-up period of 504 h (21 days) was used to compare the treatment arms. The statistical analyses were not adjusted for multiplicity.

#### **Figure S1** (**A**) Schema of the study cohorts. (**B**) Study design.

^a^The appropriateness of ensitrelvir dosing was first confirmed by the Safety Review Committee’s evaluation of the safety and PK data to Day 28 of the first 20 participants enrolled to just the 20 to <30 kg and 30 to <40 kg bodyweight categories (cohort 1); enrollment to all three bodyweight categories was allowed thereafter (cohort 2).

^b^Visits on D3 (OpV1) and D5 (OpV2) were optional; however, administration of the study intervention and participant diary recording were continued.

D, study day; OpV(*n*), optional visit *n*; PK, pharmacokinetic; R, randomization; V, study visit.

#### **Figure S2** Participant disposition.

^a^Both participants belonged to the 20 to <30 kg bodyweight category.

^b^The trial was discontinued per investigator decision because blood collection could not be performed, precluding confirmation of safety; participant burden was also considered.

^c^The reason for exclusion from the ITT population in both treatment arms was a negative SARS-CoV-2 RT-PCR result (ensitrelvir, *n*=1; placebo, *n*=2).

^d^Reasons for exclusion from the mITT population were negative SARS-CoV-2 RT-PCR result (ensitrelvir, *n*=1; placebo, *n*=2) and negative SARS-CoV-2 viral titer (ensitrelvir, *n*=36; placebo, *n*=15). Participants may have had more than one reason for exclusion.

ICF, informed consent form; ITT, intention-to-treat; mITT, modified intention-to-treat; N/A, not applicable; PK, pharmacokinetic; RT-PCR, reverse transcription-polymerase chain reaction; SARS-CoV-2, severe acute respiratory syndrome coronavirus 2.

#### **Figure S3** K–M plots of TTR of two COVID-19 objective symptoms (stuffy/ runny nose and cough plus fever) (**A**) overall, and in participants with (**B**) ≥3 and (**C**) ≤2 moderate (or worse) COVID-19 symptoms at baseline (ITT population).

COVID-19, coronavirus disease 2019; ITT, intention-to-treat; K–M, Kaplan–Meier; TTR, time to resolution.

## Table S2 Typical COVID-19 symptoms grouped as evaluated in this study

| **Number of symptoms** | **Symptoms** |
| --- | --- |
| **Two objective symptoms plus fever^a^** | 1. Stuffy or runny nose 2. Cough 3. fever^a^ |
| **Five symptoms^b^** | 1. Low energy or tiredness 2. Feeling hot or feverish 3. Stuffy or runny nose  4. Sore throat 5. Cough |
| **12 symptoms^b^** | 1. Low energy or tiredness 2. Feeling hot or feverish 3. Stuffy or runny nose  4. Sore throat 5. Cough 6. Muscle or body aches 7. Headache 8. Chills or shivering 9. Shortness of breath (difficulty breathing) 10. Nausea 11. Vomiting 12. Diarrhea |
| **14 symptoms^b^** | 1. Low energy or tiredness 2. Feeling hot or feverish 3. Stuffy or runny nose  4. Sore throat 5. Cough 6. Muscle or body aches 7. Headache 8. Chills or shivering 9. Shortness of breath (difficulty breathing) 10. Nausea 11. Vomiting 12. Diarrhea 13. Smell disorder^c^ 14. Taste disorder^c^ |

^a^Axillary temperature ≥37.5°C.

^b^These symptoms (with the exception of smell and taste disorder) were evaluated on a 4-point scale from 0 (none) to 3 (severe). COVID-19 symptoms other than stuffy or runny nose and cough were recorded as not evaluable if assessment was not possible based on the participant’s symptoms or complaints.

^c^Taste and smell disorders were evaluated on a 3-point scale from 0 (same as usual) to 2 (no sense of smell/taste).

COVID-19, coronavirus disease 2019.

## Table S3 Study eligibility criteria

|  | **Eligibility criteria** |
| --- | --- |
| **Inclusion** | - Participants aged 6 to <12 years at the time of signing ICFs by the legally authorized representative - Participants who were diagnosed as SARS-CoV-2 positive using any of the following tests of their sample collected within 72 h before randomization:   - Nucleic acid detection test using nasopharyngeal swab, nasal swab, or saliva**^a^**   - Antigen test (quantitative) using nasopharyngeal swab, nasal swab, or saliva   - Antigen test (qualitative) using nasopharyngeal or nasal swab - Participants who were randomized within 72 h after COVID-19 onset**^b^** - Participants who had at least one moderate or severe symptom (COVID-19 symptom score: 2 or 3) among the following 12 COVID-19 symptoms at screening (excluding symptoms present prior to COVID-19 onset) or those who had at least one moderate or severe pre-existing symptom (COVID-19 symptom score: 2 or 3), which was considered by the participant or guardian to have worsened due to COVID-19 at screening   - General symptoms: low energy or tiredness, muscle or body aches, headache, chills or shivering, and feeling hot or feverish   - Respiratory symptoms: stuffy or runny nose, sore throat, cough, and shortness of breath (difficulty breathing)   - Gastrointestinal symptoms: nausea, vomiting, and diarrhea - Participants who were able to orally take (swallow down) the study intervention (tablets), as determined by the investigator/sub-investigator - Bodyweight ≥20 kg at screening - Female participants were eligible to participate if they were not pregnant or breastfeeding and if one of the following conditions applied:   - Was a woman of non-childbearing potential as defined in section 10.4 of the protocol, or   - Was a WOCBP and using a contraceptive method that was highly effective as described in section 10.4 of the protocol during the intervention period and for at least 14 days after the last dose of the study intervention. The investigator/sub-investigator had to evaluate the potential for contraceptive method failure (e.g., noncompliance or recently initiated) in relation to the first dose of the study intervention - A WOCBP had to have a negative highly sensitive pregnancy test result (urine or serum as required by local regulations) within 24 h before receiving the first dose of the study intervention (see section 8.2.4 of the protocol) - If a urine pregnancy test could not be confirmed as negative (e.g., an ambiguous result), a serum pregnancy test was required. In such cases, the participant had to be excluded from participation if the serum pregnancy result was positive - Additional requirements for pregnancy testing during and after the intervention period can be found in section 8.2.4 of the protocol - The investigator/sub-investigator was responsible for the review of medical history, menstrual history, and recent sexual activity to decrease the risk of inclusion of a woman with an early undetected pregnancy - There were no requirements for contraception for male participants - Capable of providing a signed ICF through a legally authorized representative as described in section 10.1.3 of the protocol that includes compliance with the requirements and restrictions listed in the ICF/assent form and in the protocol. An assent had to be obtained directly from the participant, whenever possible |
| **Exclusion** | - Participants with SpO_2_ of ≤93% (room air) - Participants requiring oxygen treatment - Participants requiring mechanical ventilation - Participants whose SARS-CoV-2 infection symptoms were strongly suspected to worsen within 48 h after randomization as judged by the investigator/sub-investigator - Participants who were suspected of having active systemic infection requiring treatment (except SARS-CoV-2) - Participants who had or were suspected to have COVID-19-associated MIS-C/PIMS - Participants with known renal impairment and creatinine clearance of <30 mL/min calculated using the Schwartz formula or requiring dialysis - Participants with severe liver dysfunction, such as a known history of cirrhosis or hepatic decompensation (e.g., ascites, variceal bleeding, or hepatic encephalopathy) - Participants who had a history of febrile seizure or epilepsy - Participants who used any of the following drugs within 7 days before randomization:   - Approved therapeutic drugs for SARS-CoV-2 infection   - Unapproved therapeutic drugs for SARS-CoV-2 infection (e.g., interferon, convalescent plasma, monoclonal antibodies, immunoglobulins, antirheumatic drugs, corticosteroids [oral, injectable, or inhaled], ivermectin, or favipiravir) - Participants who used any of the following drugs within 14 days before randomization:   - Potent CYP3A inhibitors   - Potent CYP3A inducers   - Products containing St. John’s wort - Participants who had previously received S-217622 (ensitrelvir) - Participants who received not less than four investigational products within 12 months before randomization - Participants who participated or were currently participating in a clinical trial of an investigational product or a medical research program within 28 days before signing the ICF through their legally authorized representative - Participants who had no guardian to constantly record or verify the participant diary in an appropriate manner during the study - Participants with drug abuse - Participants who had a history of hypersensitivity to the study intervention or its components, drug allergies, or other allergies (except seasonal allergies) and thereby were judged to be ineligible as study participants by the investigator/sub-investigator - Participants who were judged to be ineligible as study participants by the investigator/sub-investigator for any other reasons |

**^a^**RT-PCR test or isothermal nucleic acid amplification method (e.g., loop-mediated isothermal amplification method or transcription-mediated amplification method).

**^b^**The time when the participant or guardian noted at least one of the 14 COVID-19 symptoms or when the participant or legally authorized representative noted COVID-19-associated worsening of pre-existing symptoms.

COVID-19, coronavirus disease 2019; CYP, cytochrome P450; ICF, informed consent form; MIS-C/PIMS, multisystem inflammatory syndrome in children; PIMS, pediatric inflammatory multisystem syndrome; RT-PCR, reverse transcription-polymerase chain reaction; SARS-CoV-2, severe acute respiratory syndrome coronavirus 2; SpO_2_, peripheral oxygen saturation; WOCBP, women of childbearing potential.

## Definitions of symptom resolution

The 12 symptoms of COVID-19 listed in Supplementary Table 2 were evaluated on a 4-point scale from 0 to 3 (0, none; 1, mild; 2, moderate; 3, severe), while taste disorder and smell disorder were evaluated on a 3-point scale from 0 to 2 (0, the same as usual; 1, less than usual; 2, no sense of smell/taste). The TTR of COVID-19 symptoms was defined as the time from the first study intervention to the time when all of the symptoms to be evaluated met the following criteria:

- Two subjective symptoms plus fever
  - Resolution of both stuffy or runny nose and cough, and axillary temperature maintained at <37.5ºC for 24 h
- 12 symptoms
  - Pre-existing symptoms that were present prior to the onset of COVID-19 and considered by the participant or guardian to have worsened at baseline (pre-dose examination). Symptoms that were severe at baseline had to be improved to moderate or better, those that were moderate at baseline had to be improved to mild or better, and those that were mild at baseline had to be maintained or improved to mild or better for 24 h.
  - Pre-existing symptoms that were present prior to the onset of COVID-19 and considered by the participant or guardian not to have worsened at baseline (pre-dose examination). Those that were severe at baseline had to be maintained or improved to severe or better, those that were moderate at baseline had to be maintained or improved to moderate or better, and those that were mild at baseline had to be maintained or improved to mild or better for 24 h.
  - Symptoms other than the above (those that were not present prior to the onset of COVID-19) had to be absent for 24 h.
- Taste disorder and smell disorder:
  - Pre-existing symptoms that were present prior to the onset of COVID-19 and considered by the participant or guardian to have worsened at baseline (pre-dose examination). Those with a baseline severity of 2 had to be improved to 1 or better, and those with a baseline severity of 1 had to be maintained or improved to 1 or better for 24 h.
  - Pre-existing symptoms that were present prior to the onset of COVID-19 and considered by the participant or guardian not to have worsened at baseline (pre-dose examination). Those with a baseline severity of 2 had to be maintained or improved to 2 or better, and those with a baseline severity of 1 had to be maintained or improved to 1 or better for 24 h.
  - Symptoms other than the above (those that were not present prior to the onset of COVID-19): a severity of 0 had to be maintained for 24 h.

## Table S4 Schedule of laboratory tests and tissue sampling

| **Procedure/Parameter** | **Schedule** |
| --- | --- |
| **AEs** | Data collected throughout the study and follow-up, from treatment initiation to Day 28 (or at study discontinuation, whichever occurred first) |
| **Laboratory tests** | Performed on Day 1 (pre-dose) and Days 6, 14, and 28 (or at study discontinuation, whichever occurred first) |
| **Vital signs** | Measured on Day 1 (pre-dose) and on Days 2, 4, 6, 14, 21, and Day 28 (or at study discontinuation, whichever occurred first) |
| **Blood samples** (PK measurements) | Collected on Days 2, 4, 5, and 6 (or at study discontinuation, whichever occurred first [optional]) |
| **Nasal swabs** (SARS-CoV-2 infection status and antiviral efficacy) | Collected on Days 1 (pre-dose), 2, 3 (optional), 4, 5 (optional), 6, 14, and 21 (or at study discontinuation, whichever occurred first) |
| **NGS** (TEAAS detection) | Conducted on swab samples collected from all participants in both treatment groups on Day 1 (pre-dose) |
| **COVID-19 symptoms** (efficacy measurement) | Recorded by participants or their legally authorized representative in a diary, twice daily until Day 9, and then once daily thereafter to Day 21 |

AE, adverse event; COVID-19, coronavirus disease 2019; NGS, next-generation sequencing; PK, pharmacokinetic; SARS-CoV-2, severe acute respiratory syndrome coronavirus 2; TEAAS, treatment-emergent amino acid substitution.

## Table S5 Definition of study analysis populations

| **Analysis population** | **Definition** |
| --- | --- |
| **Safety analysis population** | All randomized participants who had received at least one dose of the study intervention. Participants were analyzed according to the first study intervention they actually received |
| **PK analysis population** | All participants who had received at least one dose of ensitrelvir and had at least one evaluable plasma concentration. This population was used for listings and figures of drug concentrations |
| **ITT population** | All participants who were randomly assigned to the study intervention and were confirmed to be infected with SARS-CoV-2. SARS-CoV-2 infection was determined based on the result of RT-PCR of a nasal swab sample on Day 1 (pre-dose). Participants in this analysis set were analyzed according to the group to which they were assigned |
| **Modified ITT population** | All participants who were randomly assigned to a study intervention, were confirmed to be infected with SARS-CoV-2, and had a detectable SARS-CoV-2 viral titer. SARS-CoV-2 infection and viral titer were determined based on the results of RT-PCR and viral titer measurement, respectively, using a nasal swab sample on Day 1 (pre-dose). Participants in this analysis set were analyzed according to the group to which they were assigned (i.e., regardless of the treatment actually received) |

ITT, intention-to-treat; PK, pharmacokinetic; RT-PCR, reverse transcription-polymerase chain reaction; SARS-CoV-2, severe acute respiratory syndrome coronavirus 2.

## Table S6 Statistical methods used to analyze selected secondary efficacy endpoints

| **Statistical methods (for selected secondary efficacy endpoints)** | |
| --- | --- |
| 1. Pairwise comparison of the ensitrelvir and placebo arms was performed using the log-rank test 2. Pairwise comparison of the ensitrelvir and placebo arms was performed using an ANCOVA (including LSM and the difference vs placebo) 3. Pairwise comparison of the ensitrelvir and placebo groups was performed using a Wilcoxon rank-sum test 4. Kaplan–Meier curve was plotted for each intervention group 5. The median time and the 95% CI were calculated for each intervention arm 6. The difference in medians between the intervention groups and the 95% CI were calculated. The 95% CI for difference in medians were calculated by the bootstrap percentile method 7. Summary statistics (continuous variables) were calculated for each intervention arm | |
| **Secondary endpoint analysis (select)** | **Statistical method(s)** |
| Change from baseline on Day 4 in the amount of SARS-CoV-2 viral RNA | 2, 3, 7 |
| Change from baseline in the amount of SARS-CoV-2 viral RNA at each time point | 2, 3, 7 |
| Change from baseline in SARS-CoV-2 viral titer at each time point | 2, 3, 7 |
| TTR of COVID-19 symptoms (2 plus fever, 5, 12, or 14) | 1, 2, 3, 4, 5 |

ANCOVA, analysis of covariance; CI, confidence interval; LSM, least-square mean; RNA, ribonucleic acid; SARS-CoV-2, severe acute respiratory syndrome coronavirus 2; TTR, time to resolution.

# SUPPLEMENTARY RESULTS

## Table S7 Plasma concentrations of ensitrelvir (PK analysis population)

|  | **Ensitrelvir treated** | | | | |
| --- | --- | --- | --- | --- | --- |
| **Plasma ensitrelvir concentration (µg/mL)** | **20 to <30 kg**  ***N*=31** | **30 to <40 kg**  ***N*=28** | **≥40 kg**  ***N*=18** | **All**  ***N*=78** | **Adults**^2^  ***N*=943** |
| **C_24_ on Day 1** |  |  |  |  |  |
| *n* (evaluable) | 11 | 13 | 8 | 32 | 943 |
| Mean (SD) | 13.8 (4.0) | 18.1 (4.0) | 19.7 (6.2) | 17.0 (5.1) | 16.6 (3.0) |
| **C_4_ on Day 4 or C_max_ on Day 5^a^** |  |  |  |  |  |
| *n* (evaluable) | 7 | 6 | 6 | 19 | 925 |
| Mean (SD) | 30.3 (4.0) | 36.4 (6.9) | 36.4 (3.8) | 34.1 (5.6) | 26.6 (4.9) |
| **C_24_ on Day 5** |  |  |  |  |  |
| *n* (evaluable) | 13 | 14 | 4 | 31 | 925 |
| Mean (SD) | 22.8 (7.8) | 28.7 (7.8) | 25.2 (2.5) | 25.8 (7.7) | 19.5 (3.5) |

^a^Measurements taken on Day 5 are reported as C_max_ for the adult population.^2^

C_4_, plasma concentration of ensitrelvir 4 h after drug administration; C_24_, plasma concentration of ensitrelvir 24 h after drug administration; C_max_, maximum plasma concentration of ensitrelvir; PK, pharmacokinetic; SD, standard deviation.

## Table S8 Change from baseline in SARS-CoV-2 viral titer^a^ on Day 2 and Day 4 (modified ITT population)

| **Viral titer (log_10_ TCID_50_/mL)** | **Ensitrelvir arm**  ***N*=42** | **Placebo arm**  ***N*=24** |
| --- | --- | --- |
| **Baseline, mean (SD)** | 1.97 (1.01) | 1.90 (0.96) |
| **Day 2** |  |  |
| *n* | 42 | 23 |
| ANCOVA vs placebo^b^ |  |  |
| LSM (SE) | –1.28 (0.09) | –0.89 (0.12) |
| Difference in LSM (SE) | –0.39 (0.15) | ··· |
| 95% CI for difference | –0.69 to –0.08 | ··· |
| Two-sided *P* value^c^ | 0.0149 | ··· |
| **Day 4** |  |  |
| *n* | 41 | 23 |
| ANCOVA vs placebo^b^ |  |  |
| LSM (SE) | –1.43 (0.04) | –1.31 (0.05) |
| Difference in LSM (SE) | –0.12 (0.06) | ··· |
| 95% CI for difference | –0.24 to 0.01 | ··· |
| Two-sided *P* value^c^ | 0.0781 | ··· |

^a^LLOD for viral titer was 0.75 log_10_ TCID_50_/mL. LLOQ for viral titer was 1.0 log_10_ TCID_50_/mL. ULOQ for viral titer was 5.4 log_10_ TCID_50_/mL. If the viral titer was less than the LLOD, less than the LLOQ, or more than the ULOQ, the viral titer was imputed as 0.5, 0.75, and 5.5 log_10_ TCID_50_/mL, respectively.

^b^Covariate: SARS-CoV-2 viral titer at baseline.

^c^ANCOVA.

ANCOVA, analysis of covariance; CI, confidence interval; ITT, intention-to-treat; LLOD, lower limit of detection; LLOQ, lower limit of quantification; LSM, least squares mean; SARS-CoV-2, severe acute respiratory syndrome coronavirus 2; SD, standard deviation; SE, standard error; TCID_50_, 50% tissue culture infectious dose; ULOQ, upper limit of quantification.

## Table S9 TTR of COVID-19 symptoms in participants who were SARS-CoV-2 positive at baseline by RT-PCR and who had onset of COVID-19 within 72 h before randomization (ITT population)

| **Number of symptoms** | **Ensitrelvir arm**  ***N*=77** | **Placebo arm**  ***N*=37** |
| --- | --- | --- |
| TTR of two COVID-19 symptoms and fever,^a^ *n* | 68 | 32 |
| K–M estimate of TTR (h) |  |  |
| Median (95% CI) | 140.5 (109.4 to 174.1) | 146.2 (92.0 to 188.9) |
| Median difference vs placebo (95% CI) | –5.7 (–73.6 to 43.4) | – |
| Two-sided *P* value^b^ | 0.8153 |  |
| TTR of five COVID-19 symptoms, *n* | 71 | 36 |
| K–M estimate of TTR (h) |  |  |
| Median (95% CI) | 140.5 (109.4 to 168.0) | 141.3 (115.0 to 195.8) |
| Median difference vs placebo (95% CI) | –0.8 (–62.4 to 41.7) | – |
| Two-sided *P* value^b^ | 0.6093 |  |
| TTR of 12 COVID-19 symptoms, | 71 | 36 |
| K–M estimate of TTR (h) |  |  |
| Median (95% CI) | 148.9 (130.9 to 184.4) | 146.2 (115.0 to 225.3) |
| Median difference vs placebo (95% CI) | 2.7 (–73.3 to 52.1) | – |
| Two-sided *P* value^b^ | 0.5631 |  |
| TTR of 14 COVID-19 symptoms, | 71 | 36 |
| K–M estimate of TTR (h) |  |  |
| Median (95% CI) | 148.9 (130.9 to 184.4) | 146.2 (115.0 to 225.3) |
| Median difference vs placebo (95% CI) | 2.7 (–73.3 to 52.1) | – |
| Two-sided *P* value^b^ | 0.5631 |  |

^a^Fever was defined as body temperature ≥37.5ºC.

^b^Log-rank test vs placebo.

CI, confidence interval; COVID-19, coronavirus disease 2019; ITT, intention-to-treat; K–M, Kaplan–Meier; RT-PCR, reverse transcription-polymerase chain reaction; SARS-CoV-2, severe acute respiratory syndrome coronavirus 2; TTR, time to resolution.

## Table S10 TTR of COVID-19 symptoms according to number of moderate (or worse) COVID-19 symptoms (≥3 or ≤2) at baseline (ITT population)

|  | **≥3 moderate (or worse) COVID-19 symptoms at baseline** | | | **≤2 moderate (or worse) COVID-19 symptoms at baseline** | | |
| --- | --- | --- | --- | --- | --- | --- |
|  | **Ensitrelvir arm *N*=36** | | **Placebo arm  *N*=16** | **Ensitrelvir arm  *N*=35** | | **Placebo arm  *N*=20** |
| TTR of two COVID-19 symptoms plus fever^a^ | 33 | | 15 | 35 | | 17 |
| K–M estimate of TTR (h) |  | |  |  | |  |
| Median (95% CI) | 153.5 (72.6 to 336.1) | | 176.9 (56.7 to 271.8) | 140.5 (79.7 to 173.8) | | 134.0 (44.9 to 188.9) |
| Median difference vs placebo (95% CI) | –23.4 (–172.2 to 161.1) | | | 6.5 (–73.4 to 68.9) | | |
| Two-sided *P* value^b^ | 0.9102 | | | 0.6753 | | |
| TTR of five COVID-19 symptoms | 36 | | 16 | 35 | | 20 |
| K–M estimate of TTR (h) |  | |  |  | |  |
| Median (95% CI) | 144.7 (73.3 to 247.2) | | 180.7 (115.0 to 271.8) | 136.9 (104.5 to 174.1) | | 130.2 (52.2 to 150.7) |
| Median difference vs placebo (95% CI) | –36.1 (–150.3 to 101.4) | | | 6.8 (–35.6 to 78.4) | | |
| Two-sided *P* value^b^ | 0.7317 | | | 0.2878 | | |
| TTR of 12 COVID-19 symptoms | 36 | 16 | | 35 | 20 | |
| K–M estimate of TTR (h) |  |  | |  |  | |
| Median (95% CI) | 157.5 (91.6 to 247.2) | 186.4 (115.0 to 368.8) | | 143.1 (105.8 to 178.3) | 130.2 (52.2 to 203.5) | |
| Median difference vs placebo (95% CI) | –28.8 (–174.9 to 96.2) | | | 12.9 (–64.4 to 84.9) | | |
| Two-sided *P* value^b^ | 0.6601 | | | 0.2198 | | |
| TTR of 14 COVID-19 symptoms and fever^a^ | 36 | 16 | | 35 | 20 | |
| K–M estimate of TTR (h) |  |  | |  |  | |
| Median (95% CI) | 157.5 (91.6 to 247.2) | 186.4 (115.0 to 368.8) | | 143.1 (105.8 to 178.3) | 130.2 (52.2 to 203.5) | |
| Median difference vs placebo (95% CI) | –28.8 (–174.9 to 96.2) | | | 12.9 (–64.4 to 84.9) | | |
| Two-sided *P* value^b^ | 0.6601 | | | 0.2198 | | |

^a^Fever was defined as body temperature ≥37.5ºC

^b^Log-rank test vs placebo

CI, confidence interval; COVID-19, coronavirus disease 2019; ITT, intention-to-treat; K–M, Kaplan–Meier; TTR, time to resolution.

## Table S11 Respiratory pathogens detected in the study (ITT population)

|  | Ensitrelvir  n=77 | Placebo  n=37 |
| --- | --- | --- |
| Any respiratory pathogens* | 27 | 9 |
| Adenovirus | 5 | 5 |
| Coronavirus NL63 | 0 | 1 |
| Coronavirus OC43 | 2 | 0 |
| Metapneumovirus | 1 | 1 |
| Rhinovirus/Enterovirus | 12 | 3 |
| Influenza A | 1 | 1 |
| Influenza B | 8 | 0 |
| Parainfluenza Virus 1 | 1 | 0 |
| Parainfluenza Virus 2 | 1 | 0 |
| Parainfluenza Virus 3 | 1 | 0 |
| Parainfluenza Virus 4 | 1 | 0 |
| Respiratory syncytial virus | 2 | 0 |
| Mycoplasma pneumoniae | 1 | 0 |

*Including participants in whom multiple pathogens were detected

ITT, intention-to-treat

Respiratory pathogens other than SARS-CoV-2 that were tested:

Adenovirus, Coronavirus 229E/HKU1/NL63/OC43, Metapneumovirus (Human metapneumovirus), Rhinovirus/Enterovirus (Human rhinovirus/enterovirus), Influenza A/B, Parainfluenza virus 1/2/3/4, Respiratory syncytial virus, *Bordetella parapertussis* (IS1001), *Bordetella pertussis* (ptxP), *Chlamydia pneumoniae, Mycoplasma pneumoniae*

## Table S12 Proportion of participants in whom non-SARS-CoV-2 respiratory pathogens were detected over time (irrespective of the presence of SARS-CoV-2 infection; ITT population)

|  | **Total population** | | | | **≥3 moderate (or worse) COVID-19  symptoms at baseline** | | | | **≤2 moderate (or worse) COVID-19  symptoms at baseline** | | | |
| --- | --- | --- | --- | --- | --- | --- | --- | --- | --- | --- | --- | --- |
| **Analysis visit** | **Ensitrelvir arm *N*=77** | | **Placebo arm *N*=37** | | **Ensitrelvir arm *N*=36** | | **Placebo arm *N*=16** | | **Ensitrelvir arm *N*=35** | | **Placebo arm *N*=20** | |
|  | ***n*/*N*** | % | ***n*/*N*** | % | ***n*/*N*** | % | ***n*/*N*** | % | ***n*/*N*** | % | ***n*/*N*** | % |
| **Baseline** | 6/77 | 8 | 5/37 | 14 | 2/36 | 6 | 2/16 | 13 | 2/35 | 6 | 2/20 | 10 |
| **Day 6** | 8/74 | 11 | 2/36 | 6 | 1/34 | 3 | 0 | 0 | 6/34 | 18 | 1/19 | 5 |
| **Day 14** | 9/75 | 12 | 5/36 | 14 | 5/35 | 14 | 3/16 | 19 | 3/34 | 9 | 2/19 | 11 |
| **Day 21** | 16/76 | 21 | 1/36 | 3 | 7/36 | 19 | 1/16 | 6 | 7/34 | 21 | 0 | 0 |

COVID 19, coronavirus disease 2019; ITT, intention-to-treat; *n*/*N*, number of participants with respiratory pathogen detected/number of evaluable participants; SARS-CoV-2, severe acute respiratory syndrome coronavirus 2.

# SUPPLEMENTARY REFERENCES

1. Yotsuyanagi H, Ohmagari N, Doi Y, et al. Efficacy and safety of 5-day oral ensitrelvir for patients with mild to moderate COVID-19: the SCORPIO-SR randomized clinical trial. *JAMA Netw Open.* 2024;7:e2354991. <https://doi.org/10.1001/jamanetworkopen.2023.54991>
2. Ishibashi T, Shimizu R, Kubota R. Population pharmacokinetics of ensitrelvir in healthy participants and participants with SARS-CoV-2 infection in the SCORPIO-SR study. *Clin Pharmacokinet.* 2024;63:1723-1734. <https://doi.org/10.1007/s40262-024-01446-4>
